# Poaching Detection Technologies—A Survey

**DOI:** 10.3390/s18051474

**Published:** 2018-05-08

**Authors:** Jacob Kamminga, Eyuel Ayele, Nirvana Meratnia, Paul Havinga

**Affiliations:** Pervasive Systems Group, University of Twente, Enschede 7522 NB, The Netherlands; j.w.kamminga@utwente.nl (J.K.); e.d.ayele@utwente.nl (E.A.); n.meratnia@utwente.nl (N.M.)

**Keywords:** anti-poaching, conservation, surveillance, intruder detetection

## Abstract

Between 1960 and 1990, 95% of the black rhino population in the world was killed. In South Africa, a rhino was killed every 8 h for its horn throughout 2016. Wild animals, rhinos and elephants, in particular, are facing an ever increasing poaching crisis. In this paper, we review poaching detection technologies that aim to save endangered species from extinction. We present requirements for effective poacher detection and identify research challenges through the survey. We describe poaching detection technologies in four domains: perimeter based, ground based, aerial based, and animal tagging based technologies. Moreover, we discuss the different types of sensor technologies that are used in intruder detection systems such as: radar, magnetic, acoustic, optic, infrared and thermal, radio frequency, motion, seismic, chemical, and animal sentinels. The ultimate long-term solution for the poaching crisis is to remove the drivers of demand by educating people in demanding countries and raising awareness of the poaching crisis. Until prevention of poaching takes effect, there will be a continuous urgent need for new (combined) approaches that take up the research challenges and provide better protection against poaching in wildlife areas.

## 1. Introduction

**Figure 1:**
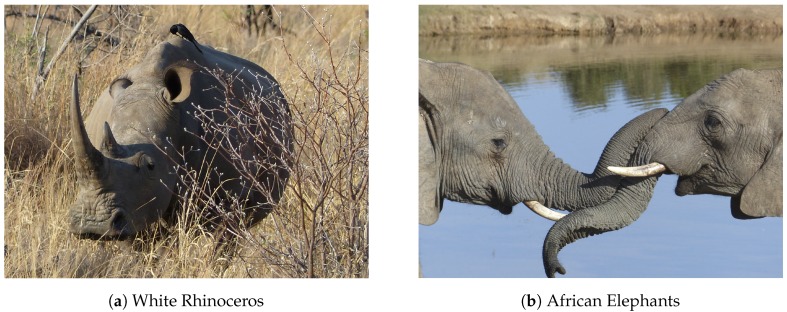
White Rhinoceros and African Elephants in their natural habitats.

**Figure 2:**
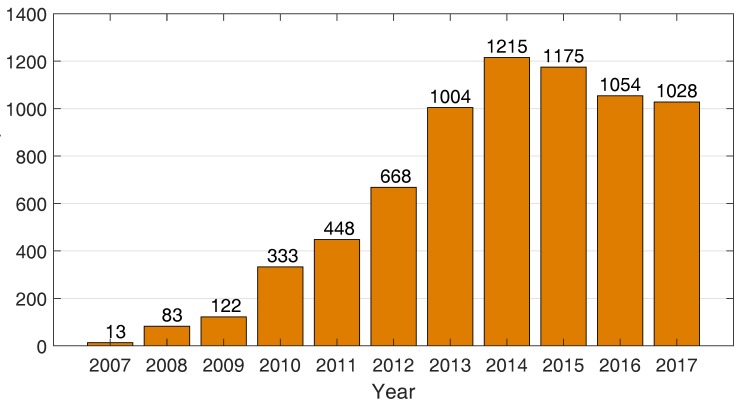
Number of poached rhinos in South Africa, adopted from the data published by the South African Department of Environmental Affairs (2017) [2].

Throughout 2016, every 8 h, a rhino was killed for its horn in South Africa alone. Moreover, an elephant is currently killed every 20 min each day. These magnificent animals are shown in Figure 1. The poaching statistics total up to 1054 rhino deaths in a population of roughly 25,000 [1,2,3] and 27,000 elephant deaths in a population of roughly 377,000 [4]. Figure 2 shows the number of poached rhinos per year. Due to increased protection efforts, the rhino poaching trend is going down again, although the losses are still extremely high. If poaching is not halted soon, the existing rhino population will not be able to procreate rapidly enough and will start to diminish once more. Between the year 1900 and today, roughly 90% of African elephants disappeared. The elephant population is currently rapidly shrinking with 8% per year continent-wide [4]. The belief that a rhinoceros horn has medicinal power, together with increasing wealth of the population, fuels the demand for rhino horns and ivory in Asian countries such as Vietnam and China [2,3]. Unfortunately, poaching is not limited to rhinos and elephants; amongst other species, tigers and pangolins are also heavily threatened by poaching for their skin, bones, and scales.

The risk that is involved with poaching is relatively low when compared to drug trafficking, yet high profits can be generated. Therefore, the trade of ivory and rhinoceros horn unfortunately remains a lucrative business for criminal syndicates [5]. Ultimately, the best solution to poaching is the eradication of demand for rhino horn, ivory, and other wildlife products [6]. Until the demand has successfully been eradicated, it remains critically important to protect the ever more fragile wildlife populations against poachers.

In this paper, we survey existing technologies that can detect poachers in wildlife areas. Because there is a large overlap with intruder detection and border patrol, we include these areas in the survey.

### 1.1. Poaching

Poaching has been around for a long time in Africa. Historically, African wildlife poachers were recruited from local communities living in close proximity to protected areas. More recently, former military personnel, police officials, or game scouts, who mostly would have had specialized training to develop tracking or shooting skills, are taking part in poaching activities [7]. Poachers with military skill often have participated in life-threatening military missions and are willing to engage in high risk activities. Other new players in the rhino poaching crisis are rogue game ranch owners, professional hunters, game capture operators, pilots and wildlife veterinarians [7]. Poachers usually receive a small part, e.g., $1000–$9000, of the horn’s black market value [8]. This is, however, still a very large amount of money for the often poor communities.

Poachers can generally be divided into three different levels [9]: subsistence, commercial, and syndicated poachers. Poachers at the subsistence level usually hunt or trap wildlife as a means to provide for themselves and their family. Subsistence poachers usually work alone or in pairs. Commercial poachers are difficult to profile. They are driven by money or poach as a ‘sport’. They may be game park managers or subsistence level poachers who were requested for specific products. Poachers at this level generally operate as a group and are frequently armed with firearms. Syndicate poachers are sophisticated and organized criminal groups, well funded, networked, and internationally orchestrated. They often operate in larger groups during the day and at night. Syndicate poachers are exceedingly well equipped with small aircraft, helicopters, assault rifles, explosives, night vision optics, vehicles, (encrypted) radio communication, dart guns and camouflage clothing. At this level, poachers have extensive skills, knowledge, and motivation [9].

### 1.2. Drivers of Demand

There is a high demand for rhinoceros horn. Vietnam and China have been identified as the main markets for rhinoceros horn and ivory in the world [7]. Three main drivers of demand can be identified as traditional medicine, trophy hunting, and opportunistic buying and gift giving.

Traditional medicine is not evidence based and remains widely used in Asian countries. This is opposite to the western medicine, which is supported by scientific methods. Vietnam, for instance, has an ancient history of using rhino horn to cure a range of illnesses. Rhino horns are made of keratin and are similar in structure to horse hooves, turtle beaks, and cockatoo beaks [10]. Rhinoceros horn is used as a ‘hangover cure’ and a palliative medicine for cancer. The healing effect of rhinoceros horn is a persistent urban myth and a main driver for the demand.

People have been hunting rhinos in South Africa for years without abusing the hunting regulations [7]. Starting in 2003, non-traditional hunters began to exploit loopholes in South Africa’s legislation. They do this to obtain rhino hunting trophies (rhino heads) to exploit for the rhino horn trade in Asia. In order to eliminate these loopholes, the South African government has implemented a number of measures. Rhino hunts are limited to one rhino kill per hunter per year and each kill must be witnessed by government personnel [11]. The country of origin of the hunter must demonstrate sufficient legislation to ensure that trophies are not used for commerce. The horns obtained from the trophy must be fitted with an Radio-Frequency Identification (RFID) chip for later identification. In addition, DNA samples of the horn should be included in the Rhino DNA Index System at the Veterinary Genetics Laboratory in Pretoria, South Africa [7].

Opportunistic buying is driven by the desire to possess exotic pets, hunting trophies, and rare plants and animals. There is evidence that the wealthy ‘elite’ uses a rhinoceros horn as a means to curry one’s favor and gain influence [7]. Rhino horns are purchased and offered as high valued gifts to political officials and other socio-economic elites within the country. Related to this is the emergence of rhino horn being accepted as a currency for payment of luxury products in certain circles.

### 1.3. Drivers of Supply

Poaching is supported by criminal and rebel groups that seek to finance their illegal activities and by professional hunters who use their experience to gain higher profit by working for international clients [12]. Unstable governments, corruption, and poor alternative economic opportunities contribute to poaching activities. Corruption facilitates the transactions between supply-, transit- and demand-countries [5].

The high payment drives young men to engage in poaching activities. Often, these people have little opportunity to find a decent job and support their families. In some communities, multiple people are engaged in poaching. In these communities, the poacher is a respected person. The poacher is a person who pays for the beer and owns a car. Cultural influence inspires other people in these communities to start poaching [5,8].

Other sources of rhinoceros horn are legally hunted trophies, privately held stocks that are not declared or registered with the authorities, or the theft of products from private and public owners and institutions such as museums [5].

### 1.4. Methods

The literature that was reviewed in this survey was found using Google Scholar (Google LLC, Mountain View, CA, USA).The following keywords were used: (i) Anti Poaching, (ii) Rhino Poaching, (iii) Elephant Poaching, (iv) Intruder Detection System, and (v) Surveillance Technology.

**Figure 3:**
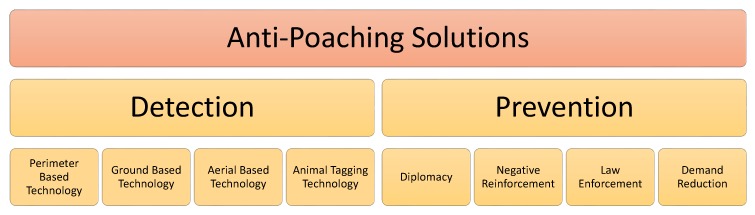
Anti-poaching methodology.

There is not a lot of literature available that focuses specifically on anti-poacher solutions. Therefore, we included material from research proposals, conservation websites, government websites, and news articles. The literature was categorized in two anti-poaching approaches, prevention and detection. The methodology of this paper is shown in Figure 3. Methods against poaching can be divided into prevention and detection of poaching. Although this survey primarily focuses on detection technologies, we will give a brief overview of the prevention of poaching.

## 2. Requirement of Poaching Detection Systems

Detection technologies often collect data from different types of sensors, which are analyzed to identify poaching incidents on time. In this section, we describe the requirements of an efficient Anti-Poaching System (APS).

- Energy Efficiency: Many locations in wildlife areas are very remote and do not have available infrastructure such as road, power lines, and network. Therefore, an APS can be isolated from power lines and rely solely on battery power and energy harvesting technologies. Any solution that is deployed in areas without infrastructure should be to operate for long periods of time to minimize costs and effort. The goal of an APS could be to have devices placed on animals or in the field, unattended, for months or years to come. Each device should be able to efficiently manage its local power supply in order to maximize the total system life-time in the long run, by deploying it on the field with a solar panel or energy harvesting mechanism to increase the energy efficiency by considering a hardware solution.
- Deployment issues: Game park managers are often uncomfortable with obtrusive technologies such as sensor poles or solar panels. These type of technologies are perceived to be unnatural. An APS usually covers a large area in which environmental factors such as weather conditions and wildlife interaction will eventually disable components of the APS. Hence, an APS should be easily maintainable so that a continuous protection of wildlife can be guaranteed. In addition, deployment of an APS should be camouflaged from poachers. Otherwise poachers might damage components and attempt to disable the APS. The system should avoid attracting visual attention, for instance, blinking LEDs, colorful mounting devices and other obviously visible pillars.
- Robustness: An APS should endure various technical and environmental deployment factors. Problems in a detection system can occur at any point between the poaching event, detection and surveillance process. For instance, the destruction of individual components should not lead to a complete failure of the overall system. Key challenges in wildlife parks include, but are not limited to, severe tropical storms, lightning strikes, flooding (near rivers) and field fires. Wildlife in parks can dig holes in the ground and play with any infrastructure that is placed in the park. Elephants and baboons, for example, can be very destructive. Hence, an APS should be robust to at least common technical faults among the distributed system components, and demonstrate strong resilience so that information remain uncorrupted.
- Scalability: The areas that are vulnerable to poaching activities are often very large. The APS should therefore be scalable. Technologically, the APS should be able to accommodate a growing number of additional devices joining the system. Scalability can be achieved by means of hardware and software techniques. When the APS is scaled up by introducing new hardware components, the system should seamlessly accept new components with no or little manual modification. Scalability also means the possibility of extending an APS in order to cover large areas, whilst staying within the bounds of other requirements denoted in this section.
- Coverage: Providing full coverage of the protected field is a very important aspect for a successful surveillance technique. In order to reduce coverage overlap, optimization methods should be used to select the best placement of the system devices. The system deployment should be positioned efficiently within a specified region to cover security blind spots and prevent intruders from exploiting these spots.
- Ethical and Legal Issues: When designing an APS that utilizes sensors located on or near animals, ethical and legal implications should be considered. When for example wildlife is collared, a general rule of thumb for the weight of an animal collar is usually 5% of their body weight [13]. Brooks et al. [14] found a significant effect of collar weight and its fit on the travel rate of zebra females. The authors compared two types of Global Positioning System (GPS) collars. Although types of GPS collars were well within accepted norms of collar weight, the slightly heavier collars (0.6% of the total body mass) reduced the rate of travel by more than 5% when foraging compared with the collar that was 0.4% of total body mass. When utilizing animals for an APS or related research, all aspects of animal handling and research should comply with methods such as those proposed by the American Society of Mammalogists for research on wild mammals [15].

Table 1 summarizes the system requirements for an APS. Even though it is practically difficult to satisfy all these mixed requirements without any compromise, an APS should attempt to comply with the most important factors.

## 3. Existing Poaching Detection Technologies

In this section, we first discuss the various types of sensor technologies that are used within an APS. A summary of the various technologies is shown in Table 2. Then, we categorize the various APSs into four domains: perimeter based technologies, ground based technologies, aerial based technologies, and animal tagging technologies. An overview of the approaches to poacher detection is shown in Table 3. We conclude this section with research challenges that were identified throughout the survey.

### 3.1. Types of Sensor Technologies

All the reviewed works in this section use various sensor technologies or a combination thereof. Therefore, in this section, we provide an overview of different types of sensors that are utilized for detection purposes along with their pros and cons.

#### 3.1.1. Radar

Radar stands for radio direction and ranging or radio detection and ranging. Radar has been used as a detection mechanism in various works [16,17,18,19,20,21,22,23]. An active radar system transmits a large amount of energy in the form of radio or microwaves with the intent to receive the reflected waves. These are then processed to determine properties of the object(s) that reflected the waves. Radar systems come in many forms with different properties that are determined by the used frequency range, line of sight, and signal processing capabilities. A radar that utilizes higher frequencies requires a lot of power and has a shorter range but will detect a target with a higher resolution. Radar systems that utilize lower frequencies use less power and have a longer range but will be unable to detect small targets. In order to detect a target, it is important that the Signal to Noise Ratio (SNR) is high enough. The SNR is determined by multiple parameters. The target only influences the Radar Cross Section (RCS) and distance to the radar device [24]. An object’s RCS value is determined by its apparent size to the radar receiver. A human being that is walking has a very small RCS, when crawling this is even smaller (10% of walking) [24]. A Doppler radar relies on the change in frequency waves when a target moves within the area of interest. High-end long range (hundreds of kilometers) air surveillance radar systems are very expensive. Depending on their complexity and range, they can cost up to millions of dollars [32]. High-end radar systems are mainly used in law enforcement, weather forecasting, military surveillance, and astronomical research [32]. In military applications, radar systems are sometimes attached to drones or high altitude airplanes and balloons in order to increase high resolution ground surveillance coverage.

Radar systems do not always require a direct line of sight with the target; however, obstacles will limit their range by adding ‘clutter’ to the radar image that makes appropriate classification difficult. Radar has the advantage that it can estimate velocity of a target and provide accurate tracking. Active radar is not stealthy because of its active nature. Radar is sensitive to interference, such as precipitation and foliage. An intruder can reduce its radar signature through careful choice of clothing, moving slowly and low to the ground [25].

#### 3.1.2. Magnetic

Magnetic sensors have been utilized in [21,26,27,28]. In general, magnetic sensors have successfully found their application in: (i) access monitoring and data tracking, (ii) archeology, (iii) detection of land mines and bomblets, (iv) detection of submarines, (v) electronic article surveillance, (vi) geophysical sciences and site surveys, (vii) home, business, and vehicle security, (viii) traffic assessment and, (ix) underwater surveillance [32].

When used as a passive technology, a magnetic sensor tries to identify a source of magnetism without generating a magnetic field. When utilized as an active technology, the sensor generates a magnetic field that is applied or inserted in materials in order to track their presence, movement or other characteristics. Metals, certain polymers, and ceramics have sufficient magnetic properties to be used in magnetic sensing technologies [32]. A magnetic dipole’s field strength decays with $1/r^{3}$, where *r* is the distance to the magnetic source. This implies that measuring a magnetic interference over large distances is very difficult and not practical for a poacher detection system in large areas. It can however be used to track vehicles along roads or persons carrying a ferromagnetic weapon in near proximity, e.g., a person carrying a gun passing through a gate [21].

#### 3.1.3. Acoustic

Acoustic sensors are usually used in the form of microphones and can sense energy signals in the audible spectrum. These can be omnidirectional or unidirectional, e.g., a shotgun- or parabolic microphone. Unidirectional microphones can detect a sound-source from a large distance. Some reviewed works have utilized acoustic sensors for the detection of animals or intruders [26,29,30,31]. Acoustic sensors have also been very popular for phone surveillance and intelligence operations [32]. Utilizing acoustic sensors in a wildlife area can provide long range sensing. Elephant rumbles that originate from several kilometres away can be detected [31], and could possibly be used as an early warning system [30]. Detecting acoustics is difficult due to the different acoustic characteristics found in different environments [30]. Another difficulty is the difference in the vocal repertoire between different species of animals.

#### 3.1.4. Ultrasonic

Ultrasonic sensors are similar to acoustic sensors but can sense signals beyond the frequency range that humans can hear. Main applications for ultrasonic sensors are sonar, industrial materials testing, and medical imaging [32]. Sonar is used for ranging and underwater detection of targets with a technique similar to radar, but the emitted energy comes in the form of ultrasonic sound signals. Ultrasonic sensors are capable of detecting most objects that have sufficient acoustic reflectivity. They are less affected by condensing moisture than photoelectric sensors. However, sound absorbing materials, such as rubber, cloth, foam and foliage absorb the sound and are hard to detect. Therefore, it becomes easy to hide from ultrasonic sensors and they do not have a practical use in detection of poachers. They have not been used in any of the reviewed works of this survey.

#### 3.1.5. Optical

The optical spectrum is visible to the human eye. Optical sensors are mostly known as cameras. Optical sensors are used widely for surveillance and detection purposes [26,33,34,35,36,37,38,39]. They are used to protect borders, inspect traffic, and people have used them in wildlife areas to monitor wildlife and detect poachers [32]. Optical sensors can have a range up to several kilometers. Classification of targets can be done by computer vision techniques or manually by surveillance personnel. Optical sensors require a line of sight with the target. Optical sensors with good range and resolution are very expensive. Thus, surveillance of a large area using this type of sensor is very costly.

#### 3.1.6. Infrared and Thermal

Infrared sensors are widely used for, amongst other purposes, intruder detection [36,37,40]. Infrared is invisible radiant energy that is a subset of the electromagnetic spectrum. The wavelengths of the infrared spectrum are just below the visible spectrum and above the micro- and radio spectrum. Infrared radiation is invisible to the human eye but can be sensed in the form of heat, for example the heat that can be felt when standing next to a fire. Anything that generates heat also generates infrared radiation. Two general categories of infrared radiation are: (i) reflected radiation (0.7–3 μm) and (ii) thermal radiation (3–14 μm) [32]. The amount of radiation emitted by an object increases with temperature. Thermal cameras detect radiation in the infrared range and transform the radiation to an image through several processes. The thermal image shows the relative difference in the amount of radiation that is generated or reflected from the objects it sees. This means that many targets could be detected with infrared sensors; however, it also means that it is very hard to detect targets depending on their environment. When a wildlife area heats up during the day, to around the temperature of a human body, the relative difference in infrared radiation from the environment and an intruder can become very small. This makes it difficult to use infrared sensors in hot environments for the detection of a person. Active infrared cameras emit infrared radiation and record the reflected energy. This approach works well to illuminate objects and persons at night but has a limited range.

Directed infrared beams can be generated by lasers and used as a trap wire [41]. A laser beam can cover a large distance and can be detected by an infrared sensor at the other end. When an intruder crosses the beam, an alarm can be triggered. Classification of the intrusion type is not possible and therefore this approach is prone to a very high false alarm rate.

#### 3.1.7. Radio Frequency

Some works utilize the effect of changes in an electro magnetic field when an intruder is crossing through [42,43]. One or two coaxial cables are buried in the ground and Very High Frequency (VHF) energy is pulsed along one leaky coaxial cable. The coupled energy is monitored from a parallel buried leaky coaxial cable. An object, person or animal that passes over the buried cable and through the electromagnetic field, which couples energy from the transmitting cable to the receiving cable, can be measured with Digital Signal Processor (DSP) techniques [20,44,45]. This type of sensing does not require a line of sight with the target. The range for this type of sensing is limited by the length of the cable, available power and quality of processing technology. This implies that this type of sensor is mostly used along a perimeter. The magnitude of the Radio Frequency (RF) field perpendicular to the cable, decays as $1/\sqrt{r}$, where *r* is the radius [44]. Commercial RF security systems state normal RF field dimensions (as seen perpendicular on the cable) of 2–3 m width, 1 m height and 0.5 m depth [46].

#### 3.1.8. Motion

Motion sensors convert physical motion into an electrical signal that can be processed. Multiple reviewed works have utilized motion sensors in the form of microphonic cable, optical cable or accelerometers [41,47,48,49,50,51,52,53]. Motion sensors are used to detect movement in fences, structures or the ground. Motion sensors are very sensitive and can be used to classify type of intrusion. Motion sensors are relatively cheap and energy efficient. The range of motion sensors is determined by the physical structure they are attached to.

#### 3.1.9. Seismic

Seismic sensors measure seismic waves generated by the impact of vehicles or footsteps on the ground. Geophones are very sensitive sensors that are used to measure seismic waves [25,26,39]. The propagation velocity of seismic waves depends on the density and elasticity of the medium they travel through. The ground supports four different types of seismic waves that each have different propagation characteristics [61]: (i) Compression waves, (ii) Rayleigh waves, (iii) Shear waves, and (iv) Love waves. Love waves travel between channels formed by layered soil. The amplitudes of shear and compression waves diminish as $1/R^{-2}$. Surface Rayleigh wave amplitudes diminish as 1/R, thus Rayleigh waves are the best type of waves to detect intruders [61]. The quality of a vibration signal heavily depends on the type of soil it travels through, thus the quality of seismic measurements is different for each environment. Loose and inconsistent soil will yield poor detection capabilities. The range for seismic detection of intruders depends on the type of soil and amount of background noise. When the SNR is 1:1, the theoretical range varies from 2–3 m with high background noise to 70–90 m with extremely low background noise [25]. Because of the physical properties of seismic waves and their high dependence on the environment, it is difficult to develop an uniform approach that can be used over large areas (with varying types of soil).

#### 3.1.10. Chemical

Chemical sensors detect and identify chemical substances or compositions of substances. Human senses are in fact chemical sensors. We can smell the air and raise an alarm when a fire is burning upwind or taste that our milk has gone sour and decide not to drink it. Animals often have better chemical sensors than humans. Dogs, for example, have very good noses and insects locate mates and food sources at great distances with their highly sensitive senses [54]. Chemical sensors come in different forms. Chemical tests are used to determine the type of substance, e.g., drugs tests. Chemical sensors also use mass spectrometry technology to detect, analyze and identify the presence of explosives and drugs. Airport security systems often use X-ray scanners in order to determine explosives inside luggage [32]. Firearms have a chemical footprint in the form of smell and substance residues, before and after use. In crime scene investigation, chemical tests are often used for research, e.g., to determine if a suspect has held or shot a firearm. Biochemical markers are used to mark potential targets so that they can be detected by special detectors. Special dyes that are used to mark banknotes in case of a robbery have been tried on rhino-horns [55] as a means to discourage poachers. This kind of dye is hard to wash off with normal washing and can be used to mark a poacher, and the horn. Chemical detectors can be used to detect the horn along the trafficking route. DNA profiling is another form of chemical sensing and is often used in crime scene investigation. The technique is also used to determine the presence and possibly the origin of seized rhino horn [7,56,57]. Chemical sensors are being put to use in very specialized areas. To our best knowledge, there currently is no sensor technology available that, for example, can smell better than a dog. Therefore, anti drug teams and bomb squads still utilize dogs in their daily practise to sniff out drugs or explosives.

#### 3.1.11. Animal Sentinels

Animal behaviours and reactions are sometimes used as detection systems. A well known example is the ‘canary in the coal mine’. Until late in the 20th century, a canary was taken into coal mines by miners to be utilized as an early-warning signal for toxic gases, primarily carbon monoxide. The birds are more sensitive than humans and would become sick, or die, before the miners would and thus act as a ‘chemical indicator’ [32]. Animal sentinels are often used in situations when (i) humans cannot always be on the alert, (ii) animals have better senses, or (iii) humans cannot safely go to places. Some reviewed works suggested to utilize animal sentinels for the detection of poachers [58,59,60]. In the natural environment, animals are used indirectly for surveillance [32]. Animals make sound calls and physical reactions when they sense danger. A barking dog or strident bird calls are sounds that are recognized by multiple species, including humans, and can be utilized as an alarm or early warning. The frenetic behavior of bees, beetles, birds, and rodents can indicate a forest fire or impending storm, while elephants can hear and feel infrasonic vibrations and know when a large animal, vehicle, earthquake, or storm might be approaching [30,31]. Hence, animal behaviour can provide an early warning that can help to detect a poacher.

### 3.2. Perimeter Based Technologies

Perimeter Intruder Detection System (PIDS) are usually deployed in the vicinity of a boundary and aligned with a barrier or linear premises [74]. When one thinks of securing a perimeter, a fence rapidly comes to mind. Developing an APS on or near an existing fence is attractive mainly in terms of power constraints. Many game parks have an existing (solar) powered fence. Fences need to be electrified in order to keep large mammals from breaking through the fence. This opens up the possibility to utilize technology that requires more power. Additionally, adding technology to existing infrastructure is non-obtrusive and pervasive.

Cambron et al. [41] propose a fence equipped with motion sensors and a laser curtain to detect poachers when crossing a fence bordering the Kruger National Park (KNP). The laser can cover segments up to 500 m and the motion sensor covers wide areas near the fence. The work does not discuss any actual classification of the intrusion event.

Wittenburg et al. [47] attached a small number of ScatterWeb [75] sensor nodes to a fence with the goal of collaboratively detecting and reporting security-relevant incidents, such as a person climbing over a fence. The sensor nodes were equipped with an accelerometer that was used to measure the movement of the fence. The authors considered the following six events as typical scenarios that a fence monitoring system may encounter: (i) kicking the fence, (ii) leaning against the fence, (iii) shaking the fence for a short period, (iv) shaking the fence for a longer period, (v) climbing up the fence and peeking over it, and (vi) climbing over the fence. The raw measurement data showed different patterns for each scenario. The sensors share information within an n-hop neighborhood and collaborate in order to distinguish a nuisance alarm from a real alarm. The authors do not discuss how well the system is able to distinguish animals that are pressing against or playing with the fence.

Yousefi et al. [48] perform similar work that classifies rattling of the fence and climbing over the fence. The hardware of the proposed system comprises a 3-axis accelerometer and a Reduced Instruction Set Computer (RISC) microprocessor. The system uses an algorithm to determine significance of the fence movement. When a significant movement is detected, the system classifies type of activity on the fence. The authors identified a difference in force patterns between climbing and rattling events and selected features that consider periodicity of the signal and relative energy between the three axes of the accelerometer for the classification. An adaptive threshold that uses information from previous frames is used to automatically adjust the sensitivity of the classifier to wind or rain.

Dziengel et al. compared their distributed detection system with four different data application scenarios with varying data processing concepts and varying network sizes to analyze the resulting communication load and the system lifetime [64]. They claim that their distributed detection mechanism makes the system more energy efficient and increases the average network life-time. The authors conclude that a relay node can achieve a highly increased lifetime when the communication to a control center can be reduced by in-network cooperation between nodes.

Microphonic cables have been used as a detection system since the 1970s. A coaxial cable produces a voltage when it is moved [76,77]. By attaching a passive microphonic cable to a fence and analyzing the voltage it produces, an intrusion event can be detected along the fence. Modern systems utilize a DSP to distinguish environmental effects from a cutting or climbing event [49,50,51]. Specialized active cables can classify an intruder and localize the point of intrusion along the cable [52,53].

More recently, optical fibre cable implementations were developed. Microphonic cable segments are usually 200 m long while optical fibre cable segment can be much longer. At the time of writing, commercial security solutions apply segments up to 1000 m [50].

Mishra et al. [62] propose the ‘virtual fencing’ concept using buried fiber optics. When someone enters the park, it is desirable to track the intruder. Multiple lines of buried fiber would be needed to estimate the direction of the intruder entering the park, thus increasing the cost significantly. Mahmoud et al. discuss the performance criteria of a real-time fence-mounted distributed fiber-optic detection system [78]. The authors present a performance analysis for different event classification algorithms.

Snider et al. [63] developed a buried fiber optic detection system and state that fiber amplifiers can extend the detection range to hundreds of kilometers. The authors propose to utilize a phase-sensitive optical time domain reflectometer to design a buried detection system. The light rays transmitted through the fiber are generated at the input by a laser diode. This laser beam propagates through the fiber along the protected perimeter. When an intruder causes vibrations in the optical fiber, it introduces phase changes in the Rayleigh backscattered light. A Field Programmable Gate Array (FPGA) system is used to analyze the backscattered light in real time to identify unusual events in the waveform. The authors implemented fiber amplifiers to extend the range of detection to cover a longer range of distance, up to 10 km. The presented work does not discuss any performance parameters.

Advantages of using fiber optic sensors in an APS include their immunity to electromagnetic interference, high sensitivity, no power required in the field (only at the processing location) and high reliability. Fence detection systems are sensitive to the trade-off between high sensitivity or a low amount of false alarms [79]. Any type of cable sensor attached to a fence is vulnerable to field fires, which often occur in dry areas like South Africa. Cable sensors attached to a fence are usually visible for intruders and can be tampered with. This makes a cable sensor on a fence less stealthy. Tampering can be detected and localized, but repairing can be costly.

Kim et al. created a prototype of a wireless sensor based system for perimeter surveillance [26]. They developed the system on ANTS-EOS (An evolvable Network of Tiny Sensors—Evolvable Operating System) [65] architecture, which is adaptive to changing network conditions. They integrated sensors located on a fence and on the ground with a mobile robot, an Unmanned Aerial Vehicle (UAV), and a visual camera network to monitor the fence. All sensor-nodes were installed with acoustic, magnetic, and Passive Infrared (PIR) sensors to detect intruders. Seismic sensors were installed on the ground nodes and piezoelectric sensors on the fence nodes. The authors propose an auto-adapting base threshold that changes based on the standard deviation σ of a sensor’s output signal. The real threshold to signal an event is the base threshold plus 2σ. When the energy of a sensor is above the real threshold for a longer period, the node will signal that an intruder has been detected. The Unmanned Ground Vehicle (UGV), UAV, or a static camera was then used to verify the intrusion and/or track the intruder. The system seems to be very complex when it is scaled up due to the large amount of different sensors that have been used in this approach.

Rothenpieler et al. designed a networked system with infrared sensors that is able to detect an intrusion event [40]. The system alerts and triggers an alarm when an intruder is detected crossing the perimeter. The authors developed an algorithm to first detect any unusual movement across the perimeter and then fine-tune the triggered signal locally in the network to eliminate false alarms and eventually sending the alarm to central authority. They present simulation results of networks containing 200 and 2000 nodes and compared this with the results of their first prototype network that contained 16 nodes.

Aseeri et al. [33] discussed a method to improve data security in small and energy efficient Wireless Sensor Network (WSN)s that are used for border surveillance. They argue that information collected from WSNs is crucial in making border surveillance decisions. They simulated a distributed sensor network and analyzed possible attacks such as sensor destruction or signal jamming. In this work, the authors present a neighbouring peer, trust based communication model that can maintain a high level of security in a WSN.

He et al. designed and implemented an energy-efficient surveillance WSN [27,28]. Their system allows a group of cooperating magnetic sensor devices to track the positions of moving vehicles. They evaluated the performance of their system on a network of 70 MICA2 motes equipped with dual-axis magnetometers, distributed along a 85 m long perimeter, on both sides of a grassy path. From experiments, they determined that these magnetometers can sense a small magnet at a distance of approximately 30 cm and a slow moving car at a distance of approximately 2.5–3 m. The authors tackled the trade-off between energy efficiency and surveillance performance by adaptively adjusting the sensitivity of the system. The key parameter they use to do this is the Degree of Aggregation (DOA), defined as “the minimum number of reports about an event that a leader of a group waits to receive from its group members, before reporting the event’s location to the base station”. Increasing the DOA leads to less false alarms and messaging overhead but increases the reporting time from a local cluster to the gateway. Thus, an optimization problem is to find the optimal DOA to achieve an acceptable report latency whilst minimizing the messaging overhead and false alarms. In their work, they did not consider the impact of the sensor node sleep time.

Sun et al. introduced BorderSense [39], which utilizes sensor network technologies, including wireless multimedia sensor networks and wireless underground sensor networks. BorderSense is a system that coordinates multiple technologies such as unmanned aerial vehicles, ground sensors, underground sensors, and surveillance towers equipped with camera sensors to detect intruders.

### 3.3. Ground Based Technologies

Ground based technologies can detect intruders over a large area and do not have to be constrained to a linear detection zone. They can detect a person or vehicle intruder entering and/or moving within a defined detection zone, ideally with tracking capability to monitor the direction of single or multiple intruders [74]. Such technologies usually utilize volumetric sensor technologies that cover a large usually omnidirectional area rather than a linear detection zone. The detection zone can have a radius ranging from tens of metres to a few kilometres. An intrusion alarm is triggered by entering the zone or moving within it. Ground based technologies can be camouflaged to intruders and provide a high level of stealth. Sensors can be buried a few centimeters below the surface. Because these types of systems are usually hidden, they are often used where a fence would be considered to be obtrusive. Wildlife such as warthogs dig a lot of holes, especially underneath fences [80]. This means that a buried system in a wildlife park has a higher probability of being damaged.

Coaxial cables have been utilized as an ‘invisible’ RF intrusion detector, originally referred to as ‘leaky cables’ [42,43]. One or two coaxial cables are buried in the ground and VHF energy is pulsed along one leaky coaxial cable. The coupled energy is monitored from a parallel buried leaky coaxial cable. An object, person or animal that passes over the buried cable and through the electromagnetic field, which couples energy from the transmitting cable to the receiving cable, can be measured with DSP techniques [20,44,45]. This technology has been developed since the 1970s and is commercially sold for applications such as military and airport security systems. The method was elaborated by the original authors into a guided Ultra Wide Band (UWB) RADAR system [20].

Souza et al. [66] proposed a WSN based framework for target tracking under wildlife for the purpose of event detection in their territorial habitats. They divided the field area into clusters of networks by deploying fixed sensor nodes on the ground to predict the path followed by the target being tracked. Their system detects animals then computes their current location and predicts the next possible position. The main data learning and processing is performed centrally. Due to low reliability in the sensor data collection and energy limitations, implementation of this solution by only using clustered wireless sensor networks have been a challenge to real-time applications.

Zeppelzauer and Stoeger propose an automated early warning system that can detect the presence of elephants [30]. Langbauer et al. conducted 58 playback experiments with free-ranging elephants in Namibia [31]. They estimated the distance over which some of their low-frequency calls are audible to other elephants. They recorded a full response from the elephants for distances up to 2 km. Their results were consistent with the hypothesis that the very low-frequency calls of elephants function in communication between individuals and groups of elephants separated by distances of several kilometers, up to 4 km. The method detects elephant rumbles that originate from several kilometres away. They combine this with the visual detection of elephants in video footage. The proposed method by Zeppelzauer and Stoeger [30] can help in the human–elephant conflict in Africa and Asia by providing an early warning so that proper counter measures can be taken. It may be possible to apply the technique in an APS. When it is possible to understand an elephant’s warning signal, it can act as a long-range anomaly indicator. The authors do point out that detecting acoustics is difficult due to the different acoustic characteristics found in different environments. Another difficulty is the difference in the vocal repertoire between different species of elephants.

Suman et al. [29] demonstrated the use of acoustic signal based gunshot and other alarming animal sounds to detect poachers in the wild. The authors implemented the Mel-Frequency Cepstrum Coefficients (MFCC) [81] power spectrum of sound based on linear transform to extract and learn the signal. They have a learning phase to build a table of known sounds that can be matched in real time by a classification algorithm.

Mishra et al. used sound and light intensity sensors to detect an intruder passing a perimeter [35]. They demonstrated that an artificial neural network enables intruder detection with relatively simple sensors. They studied and learned the intrusion events offline and trained the system about the patterns of the intruder movement to help predict and analyse the intrusion incident in the future. Their algorithm was implemented and tested on 32 MICAz devices.

Arora et al. [21] proposed a wireless sensor network with metallic object detection capabilities. The sensor nodes utilize magnetometers and micro-power impulse RADAR sensors. Metallic mobile intruders such as vehicles are detected. They use pulse Doppler sensors as their RADAR platform. These sensors can identify the intruder from up to 18 m distance. The sensor nodes cooperatively network among each other, to intelligently decide if the mobile event is metallic or non-metallic. The authors tested their performance in a confined perimeter within an area. To accomplish this task, the authors recommend that accurate periodic time synchronization should be maintained among the individual sensor devices. They propose using a master node that would be responsible to send periodic synchronization values. The authors implemented the so called ‘influence field’, which represents the sensor nodes that simultaneously hear the moving intruder or object and autonomously predict the pattern of movement.

He et al. [23] described their current implementation of networked eMoteNOW sensor nodes that utilize BumbleBee micro-power RADARs in [22]. These RADARs have an omnidirectional sensing range of 10 m. The implementation is being used in an ongoing human and wildlife protection WSN in the Panna Tiger Reserve in Madhya Pradesh, India.

The networked, RADAR-equipped, motes were deployed in an array to form a Virtual Fence (VF) that classifies animals foraying out of the forest, as well as humans trespassing into the reserve. The VF and Activity Recognition Monitor (ARM) are connected by a grid of relays to the nearest base station, which is typically located at the nearest guard station. The system is able to distinguish moving bush and trees from actual targets of interest. The approach is not yet very scalable for large areas, as each RADAR only covers a radius of 10 m.

Zhang and Jiang et al. utilized an UltraWide Band (UWB) WSN to detect intruders in forested areas [67,68]. Their work shows the potential to use off-the-shelf UWB transceivers or existing Wireless Sensor Networks (WSNs) to detect an intruder. They proposed utilizing a combination of Principal Component Analysis (PCA) [82] coefficients and the channel characteristic parameters from the received waveform to detect intruders. This resembles a passive RADAR on the receiving end. The authors used a Support Vector Machine (SVM) [83] based classifier to classify the type of intrusion. They claim that the proposed system distinguishes an armed from an un-armed intruder through analyzing the effects of metal on electromagnetic waves. Through extensive practical evaluation of their system, the authors claim their method to be efficient, accurate and robust in identifying the target in dense forests.

### 3.4. Aerial Based Technologies

Mulero et al. [36] deployed drones equipped with heat sensing and camera devices to pinpoint poachers in the vicinity of a national park [37]. They performed several tests to observe the effectiveness of the proposed technique to prevent poaching incidents. The tests proved to differentiate people from rhinos or other animals at altitude ranging from 100–180 m. However, this solution has its own limitations. It will not be able to clearly scan densely populated forests. Detection with regular cameras is only practical during day time. The running cost is relatively high since the drone has to make a number of flights per day. This limits its applicability to real-time low cost monitoring solutions. It can however be used as a supporting surveillance tool for other more efficient techniques to monitor the area, and if needed to confirm the poaching event visually.

Park et al. [70,71] developed an Anti-Poaching Engine (APE) that coordinates air surveillance with rangers on the ground using predictive analytics. They combined a mathematical model of poachers’ behaviour with a model that describes the animals’ movement pattern. The authors developed scalable, geographically sensitive algorithms that can be used to automatically fly a set of drones and coordinate them with a set of ranger patrols on the ground. The APE uses behavioral data from poachers derived from previous poaching incidents. The coordinated ground-flight patrols take terrain information into account (elevation data and road connectivity). In order to place as much animals as possible within the action range of multiple anti-poaching patrols, the APE generates an automated flightpath for the drones to fly on autopilot and optimal locations for the anti-poaching units to patrol. The APE is implemented in a game park in South Africa as an initiative named “Air Shepherd” [38]. There is always one drone airborne surveying the area. Batteries of drones are quickly changed and the drone re-launched. Thermal camera’s on-board the drones stream live data back to a van, in which controllers continuously monitor the feed and report anomalies to anti-poaching patrols in the area. Air Shepherd claims that, since they started the initiative in this particular area, poaching events went down from 19 per month to 0 [38].

The Wildlife Conservation UAV Challenge [69] aims to design low cost UAVs that can be deployed in wildlife parks and are “equipped with sensors able to detect and locate poachers, and communications able to relay accurate and timely intelligence to Park Rangers”. Several teams from all over the world take part in this challenge and are developing UAV aided anti-poaching solutions.

Aerial vehicles or ‘drones’ are very agile and can cover large areas. They are however very vulnerable and easy to shoot out of the sky. Black colored, electric powered fixed-wing aircraft are more difficult to shoot out of the sky at night. However, with night-vision equipment and a shotgun that has the correct choke settings, a poacher could probably shoot down these drones when they really want to, thus rendering an approach using aerial vehicles more delicate. Drones are also obtrusive for people living in wildlife areas, or tourists visiting the wildlife park. Low flying UAVs disturb the experience of being in the wild. Malfunctioning drones can be dangerous with a chance of crashing into animals or people.

### 3.5. Animal Tagging Technologies

Animal behaviours and reactions can be used as detection systems (see Section 3.1). One approach to capture animals’ reactions to their environment is by tagging or attaching sensing devices directly to their body. Tagging is used to monitor the changes in the animals’ body or movement behaviour. Recently, several efforts are proposed to use tagging as part of an intrusion detection system.

In 2007, Yasar et. al. [58] proposed a Mobile Biological Sensor (MBS) based system, utilizing animal behaviour to assist in early detection of forest fires. The main idea presented in this paper was to utilize animals as sensors by tagging them with body sensor devices to detect their behavioural changes. The animals used in this detection system are animals that are native animals living in the forest. The attached sensors (thermo and radiation sensors with GPS features included) measure the temperature and transmit the location of the animals. In this work, they primarily propose two different detection methods: Thermal Detection (TD) method to measure instant temperature changes, and Animal Behavior Classification (ABC) method to classify sudden changes in the animals’ behaviour. Thermal Detection (TD) is essentially based on the idea that the animals, especially reptiles, know how to escape fire. In this method, thermal and radiation data obtained from the MBSs’ is classified and evaluated to determine whether a forest fire has occurred or not. Animal Behavior Classification (ABC) is based on the idea that the fire creates panic on the animals movement, especially mammals. Each mammal in a group instinctively tries to be closer to their herd; however, in case of fire, panic animals try to disperse in random direction unpredictably. Thus, such observations on the behavior of animal groups can be used for behavioural classification. The system uses wireless access points to relay data to the central computer system, which further classifies actions of the animals. Continuous panic in the MBSs shows that a problem with the animal is occurring and should be investigated.

Similarly, in a concept paper, Banzi et al. [59] suggested utilizing wild animals’ behaviour to stop poaching. They propose protecting elephants from poaching by attaching an appropriate sensor on their body with a visual, IR camera and GPS. In their concept, access points received MBS locations continuously and sent it to a central computer where it was stored in a database. A classifier continuously indexed to a central database to determine any abnormalities in the behaviour of the MBSs. Using artificial intelligence tools, a classifier attempted to determine whether or not there were abnormalities in animal behaviour. A sudden panic of animals caused an abrupt change in the graph of a classifier in the central computer; this showed a potential incident and the system responded by first rising an alarm, and then displaying the current GPS location. Eventually, the Anti-Poaching System (APS) will display the triggered alarm by processing the received event with different techniques such as edge detection, thresholding and filtering to ensure that Anti-Poaching Team (APT) are getting the correct data. Furthermore, when a disturbance within the animals is detected, the system will send out an automated alert message to the APT. If immediate measures are taken by the game rangers poachers will be arrested and poaching will be eliminated in this way. A challenge with this concept emerges when a considerably large number of animals are to be monitored. A lot of animals need to be tagged, which requires the capture and sedation of the animals. It creates a large routing overhead; therefore, high latency, and also a high cost of deployment for tagging individual animals in the park, possibly limiting its applicability to large scale cases.

Paul et. al. [72] proposed a novel tracking method that could be used to implement real-time APS. They designed an APS module fitted with miniature devices to detect a poaching incident and exactly locate the poaching event and the rhino positions in real time. A camera device is implanted in the front lobe of the rhino’s horn. Multiple body sensors continuously monitor the physiological behaviour of the animal. When an on going active poaching event is sensed, the GPS device sends the exact location of the Rhino. Meanwhile, data about the poaching event is directly sent to the central system to alert the rangers to arrive at the poaching location in the hopes of saving the rhinos. Such systems will significantly increase the chances of successfully catching the poachers. In combination with other APS techniques, this approach could lead to a more robust APS solution to fight the poaching schemes. However, this kind of APS is non-preventive, meaning it usually does not save the rhinos before being poached to death. However, it will help in the criminal prosecution of the poachers by keeping a video record. Locating the rhino’s position might be dangerous. Because of corruption, this valuable information might fall in the hands of the poachers themselves.

Recio et al. [60] also demonstrated the application of GPS tagging of wild animals to track the different behavioral changes in animals, mainly to assess the main environmental, technical and behavioural causes of error in lightweight GPS-collars suitable for medium to small terrestrial mammals. GPS tracking allows localization of animals in areas where there is low accessibility to other infrastructures. It does not prevent poaching incidents but is mainly meant to track the animals’ position.

**Figure 4:**
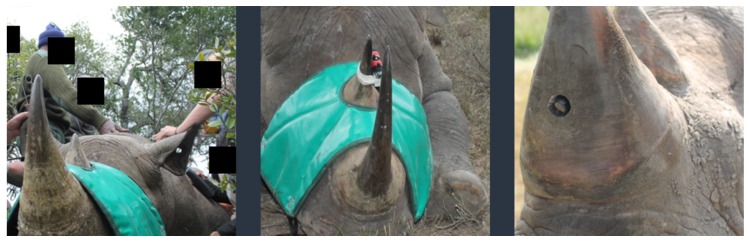
The camera component implanted in the front lobe of the rhino’s horn [34].

A GPS implementation is further extended in Project RAPID [34]. The researchers have demonstrated the usefulness of GPS in rhino poaching prevention with GPS communication to track down poaching incidents and alert the park rangers. They also installed a camera system on the horn to capture the poaching event for criminal conviction purposes (Figure 4). GPS satellite collars and Very High Frequency (VHF) radio techniques have been combined to improve the tracking and monitoring capability of GPS systems [84]. However, these solutions incur the low communication reliability and data update accuracy problems of the conventional GPS systems integration. The authors use the satellite to relay real-time information and monitored events to the central system for analysis. The high latency or response time associated with the communication makes it difficult to prevent the poaching incident before it happens. The legal and ethical issues involved with drilling the horn to implant the visual camera also hinders its practical implementation in real world scenarios (Figure 4).

An alternative collar based solution is proposed by Intel (Santa Clara, CA, USA), as a contribution to a conservation effort in the Madikwe Conservation Project [73].A kevlar-based ankle collar was attached to the rhino’s ankle during a pilot project with five black rhinos. The collar contains Intel’s Galileo board that is used to track the rhino’s location and can communicate through a 3G connection. A durable solar panel is attached to the collar to recharge the batteries. A RFID chip was placed inside the rhino’s horn. When the RFID chip is out of reach of the Galileo board, an alarm is triggered and APTs are alerted via the 3G connection. They can then rush to the scene with helicopters, drones or other vehicles. This approach requires tranquilizing the rhino to attach, repair or remove the collar, and, each time, the RFID grows out of the horn. This system is only helpful to try and catch the poachers. It does not save the rhino from being shot directly. It can be argued that the improved poaching detection causes more fear of being captured and will eventually stop more poachers from any poaching activity.

### 3.6. Research Challenges and Future Directions

In this section, we discuss open research challenges and future directions for the improvement of anti-poaching methods. Throughout the surveyed works, it becomes apparent that currently no Anti-Poaching System (APS) or sensor technique is able to meet all the requirements for effective poaching detection by itself. Poaching detection is a very difficult challenge; moreover, it is very critical to find effective solutions rapidly, as elephant and rhino populations are diminishing at an alarming rate.

#### 3.6.1. Sensitivity and Reliability

None of the reviewed work provides the high Probability of Detection (POD) and low response times that are needed for park rangers to intervene before the animals are shot to death. Increasing the sensitivity and responsiveness of an APS remains a general open issue. However, high sensitivity easily generates false alarms and avoiding this is crucial. Developing a reliable large scale APS with a good POD to False Alarm Rate (FAR) ratio remains an important open research challenge.

#### 3.6.2. Sensor Development

As we have seen throughout the survey, the many challenges in poaching detection cannot be tackled through individual sensor techniques. Therefore, the sensor community should not focus only on improving individual detection sensors systems. We argue that sensor development techniques in anti poaching methods can be improved by considering a higher level of sensor technology in the form of distributed *cognitive* sensor systems. We coin the term *cognitive sensor systems* because current day technology allows us to create sensory systems that are not only capable of perception, but also learn over time as more data is collected. Cognitive sensor systems can be seen as the state of the art in Wireless Sensor Networks (WSNs) [85] and inherit all its properties.

An approach with combinations of multiple sensor technologies and processing techniques promises to be a lot stronger and efficient as we will emphasize below. For most sensor systems, high spatial and temporal resolution is a very costly requirement because they scale up poorly or are too slow for the large conservation areas. Developments in the areas known as ‘big data’ and Machine Learning (ML) have taught us that each sensor does not have to be of very high quality—which are often very expensive—many sensing data from cheap, less accurate, sensors can be combined and provide a good sensing quality over large areas through big data techniques [86]. The implementation of ML techniques in sensor systems has to be investigated to create truly cognitive local senor systems and eventually a cognitive APS. The harsh environment and potential tampering by poachers will cause components of an APS to fail; thus, a high resilience is demanded. Distributed systems have proven to be very resilient because they are capable of self organizing [87]. In other words, when a component fails, the system can automatically reorganize itself. In a distributed APS, each sensor-node itself should be cognitive. It should not merely sense the environment, but make decisions locally on the platform and together with other sensors in the neighborhood. For example, the lifetime of a wireless sensor that runs on a battery can be extended by transmitting only data when it is perceived as important. Additionally, when data is transmitted over longer distances, energy of multiple nodes (the collective) can be saved when sensors cooperate and group data from sensors in the neighborhood through a low power short range radio prior to sending the combined data over a longer distance with a higher power, long range, radio. More and more data is collected over time when an APS is deployed in the field. The latest ML techniques should be investigated to exploit the knowledge of the collected data so that the effectiveness can be increased over time, creating a truly cognitive sensor system.

In summary, we argue that the development of cognitive sensor systems, through the combination of multiple sensor technologies and ML techniques, will be able to tackle the challenges found in effective poaching detection.

#### 3.6.3. Logistics

Throughout the surveyed works, it becomes apparent that high spatial and temporal resolution in monitoring are required for effective poaching detection. In other words, the detected location should be accurate enough for rangers to be able to locate the poachers and the event has to be reported in a timely manner—before the animal is killed. Effective covering of the surveillance area is discussed in some of the research works presented. For example, utilizing RADAR nodes that overlap in range will provide 100% coverage. However, RADAR is very expensive. The development of a system with large coverage, which remains affordable and requires (minimum) non-obtrusive infrastructure, remains a challenge. In designing an APS, there is always a need to accommodate a growing number of wild animals that need protection or an increasing surveillance area that needs coverage. Therefore, scalability remains an open research challenge.

Most works did not discuss deployment issues in proposing their APS approach. Even though deployment issues are a multifaceted problem and not easy to tackle, issues such as camouflage and the impact of obtrusive technologies are often not discussed in many approaches. The utilization of existing infrastructures such as pillars near lodges, fences (usually electric), high sites (phone masts), and roads to realize an APS have not been discussed and remain a challenge. In future research, we recommend that researchers discuss their work and proposed solutions in relationship with deployment issues.

Furthermore, we observed that reviewed works often ignore the implementation, running and maintenance costs. These costs might render an APS unaffordable especially for impoverished countries. Since many of the endangered animals are in under-developed parts of the world, deployment cost still remains an issue. For example, in the case of aerial surveillance [70,71], when a longer surveillance time is required, the drone equipment will be bigger and more expensive than the smaller flight-time drones (30–90 min). Such aerial surveillance systems that utilize thermal cameras need larger drones with high performance battery supply [36,37,70,71].

#### 3.6.4. Legal and Politics

Existing legislation suffers from loop-holes and corruption that can be abused by poachers and organized syndicates to continue their criminal activity [8]. Political issues to stop poaching and produce effective legislation remain a challenge. Likewise, information confidentiality is not often discussed in the reviewed works [69,70,71], while it is very important in countries where corruption and bribery are big problems. A solution to prevent corruption could be the implementation of an hierarchical management system in the APS. In such a system, a small amount of responsible personnel should be made aware of delicate information and the personnel that is exposed to this information should be thoroughly investigated for their credibility.

## 4. Prevention of Poaching

The eradication of poaching is tremendously challenging. However, it has been shown in recent history that it is possible to stop the poaching crisis at the source [88]. The previous rhino poaching crisis in Africa took place between 1970 and 1993. At this time, Taiwan was the world’s largest consumer of rhino horn. Under pressure from the international community, Taiwan banned the trade and strongly enforced this ban, which resulted in negligible rhino poaching between 1994 and 2008 [88].

In this section, we discuss prevention of poaching in four disciplines: (i) diplomacy, (ii) law enforcement, (iii) negative reinforcement, and (iv) demand reduction.

### 4.1. Diplomacy

Diplomacy to stop poaching is orchestrated through organizations and governments. The Convention on International Trade in Endangered Species of Wild Fauna and Flora (CITES) [89] is an international agreement between governments. Through this agreement, governments’ attempts to stop illicit wildlife trafficking on multiple levels and ensure that the international trade in specimens of wild animals and plants does not threaten their survival [89]. Countries adhere to the CITES agreement voluntarily. The CITES provides a framework that is to be respected by each country, which should adapt its local legislation to ensure that the agreement is implemented at a national level [89]. TRAFFIC [90] is a strategic alliance of the World Wildlife Fund (WWF) and International Union for Conservation of Nature (IUCN) and draws on the expertise and resources within both organizations. A major part of TRAFFIC’s program is working closely with law makers, law enforcers, and the court system to make sure that appropriate laws are in place, fully understood by those enforcing them and violators receive appropriate penalties [90].

Private game parks constantly acquire horns from animals that died from natural or ‘management-related’ causes, from poached animals or from seizures [7]. Horns that have been acquired through one of these methods must be registered with the government and handed over to the national rhino horn stockpile, which utilizes a database developed by traffic. The WWF and traffic fight for stronger penalties, effective enforcement, cross-border and inter-agency collaboration, and a disruption of criminal networks. In 2014, traffic and WWF launched the Wildlife Crime Initiative (WCI), which generated high momentum and high-level political will [91].

### 4.2. Law Enforcement

In order to prevent poaching officers from being stationed inside wildlife parks to perform patrols and pursue poachers, Haas et al. [92] propose an interdiction and pursuit solution in the form of a tool that generates optimal patrol routes. Interdiction patrols attempt to place officers at the location of an attempted crime. Besides interdiction patrol routes, the proposed solution attempts to generate optimal routes to intercept fleeing suspects. When signs of poachers are found, patrol officers begin the pursuit by following a route that is generated by the tool. The proposed routes can be updated as new information is given from the field. The tool is based on Stackelberg game theory and represents different types of poachers and their utilities. The tool has been evaluated through simulation and by implementation of a prototype in a wildlife reserve to test its performance with actual rhino poaching incidents. The authors demonstrate the capability to support the search for poachers within a reserve. In its current form, the system does not take into account the impacts of parameters such as time-of-day, moon phase, and terrain-type on the utility of a candidate route to the attackers. The tool only depends on the history of routes followed by the previous poachers, which could be problematic to predict future incidents in real time. Like Fang et al, discussed below [93], Haas et al. state that poachers often return to the areas where they were successful before. This spatial data, together with the targets’ wildlife spatial behavior, improved the performance of the system proposed. In order to achieve good real-time updates, the tool depends on good communication between officers in the field and the central system.

Fang et al. [93] report on the key technological advances that evolved a decision aid, proposed in 2014, to a regular deployed application called PAWS. PAWS is a game-theoretic application that was deployed in Southeast Asia and attempts to optimize foot patrols so that poaching can be prevented. PAWS incorporates multiple features to generate a route for the patrol (patrol strategy). Features that are used include terrain information (e.g., lakes and drainage), contour lines (elevation information), previous patrol tracks, base camp locations and previous observations (animal and human activity distributions). The authors learned that it is important to visualize the results of the algorithm for communication and technology adaption by patrollers. Additionally, they learned that patrollers prefer having a small device that can be used to collect patrol data and to show the suggested patrol routes.

In another work, the authors present CAPTURE [94], a more recent model to predict poaching activities. In the newer model, the authors take into account the dependence of the poachers’ behaviors on their activities in the past and the probability that rangers will actually find signs of poaching in an area. The dataset used for training of the CAPTURE contains more features than for previous models. The authors learned feature weights through 12 years of data collected in the Queen Elizabeth National Park (QENP) in Uganda. From these feature weights, the authors inferred that poachers tend to avoid regions with higher patrol coverage and go back to areas where they have poached before. The authors were surprised that animal density was not a good indicator for the prediction of poaching.

Kar et al. [95] build on the aforementioned approach and propose a simpler, decision tree-based, model named INTERCEPT. The authors argue that CAPTURE cannot actually be deployed because it suffers from critical limitations such as poor performance. INTERCEPT significantly outperformed the more complex CAPTURE model. Because INTERCEPT is a decision tree based model, it is easier to understand the generated patrol strategy. INTERCEPT has been deployed for a month in the QENP and an active elephant snare (trap) was removed, plus materials to make more snares, potentially saving the lives of multiple elephants. The authors are planning to incorporate INTERCEPT into PAWS [93].

Sintov et al. [96] argue that the success of new technology hinges on user adaption. The authors conducted a case study that focused on PAWS [93] in order to understand users’ adoption decisions and how to account for these so that the introduction of new technologies to rangers can be better streamlined. The authors argue that little is known about factors that contribute to the adoption of conservation technology. Data was collected through surveys that were completed on the final day of a 3-day workshop by people who use PAWS in the field. The case study was rather small with 29 participants and due to the cross-sectional design the results are not causal. The authors found that program engagement by the users is positively associated with adoption intentions and perceived usefulness. It was shown that more resistant rangers are often younger. The work provides evidence that educational interventions can provide benefit to the introduction of conservation technology. In general, the works shows that it is important to include social sciences in conservation efforts for an optimal effect.

Agreements and laws are only effective when enforced by the governments properly and sufficiently. The situation in Taiwan [88] has shown that proper enforcement in countries that consume rhino horn can result in a complete demand eradication. Enforcing law by ‘militarizing’ wildlife parks can, however, backfire on the intended purpose. Green Militarization [8] is a new term to describe a recent trend around the world, which uses military and paramilitary personnel, training, technologies, and partnerships in pursuing conservation efforts. Military skills and soldiers have been utilized to forcibly evict communities to create, maintain, or expand protected areas. Because of this, these communities hold a growing grudge towards general conservation efforts. Park rangers used to have a good relation with local communities bordering KNP, which proved useful for conservation. Now that rangers are often armed and sometimes shoot people with lethal consequences, local communities do not trust them as much and the fruitful relation between the two parties has eroded. The situation is even more complex on the Mozambican side of the KNP [8]. Communities living in areas bordering the KNP on the Mozambican side have been cut off from the KNP and are not sharing in its economical benefits. On top of this, family members in the community are sometimes (assumed to be) poaching in the KNP and shot on sight. It is these communities that should have a good relation with conservation parties to identify poaching activities. Lunstrum argues that this effect makes it easier for criminal syndicates to solicit people from local communities for poaching activities and is an example of how militarization can backfire against its original intended purpose [8].

### 4.3. Negative Reinforcement

Negative reinforcement aims to discourage poachers from killing animals. Some of the approaches to negative reinforcement are obtrusive to the endangered animals because they involve direct physical contact with the animal’s body. All obtrusive techniques require periodic capture and full sedation of the animal that needs protection.

**Figure 5:**
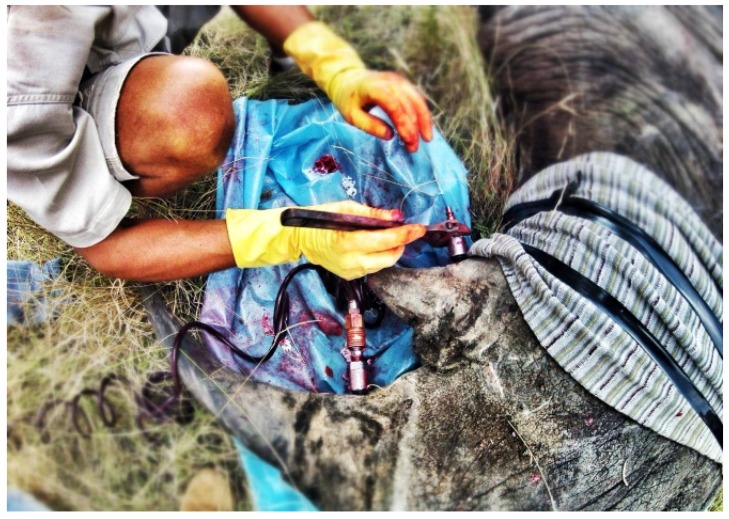
A snapshot of dyeing and removal of the rhino’s horn in an attempt to stop poaching. The horn is poisoned with chemical to make the horn useless to consume [98].

In an attempt to discourage poachers, the horns of live rhinos have been injected with pink dye and poisonous chemicals to render the horn unusable. As shown in Figure 5, the intoxication process is done by drilling a hole directly into the horn. The chemical should easily be detected at airport check points during transportation to make it difficult to illegally transport the horns. The poison can seriously hurt the health of unknowing end-users. Moreover, in a recent study, Ferreira et al. [97] refute claims that dyeing horns is effective. Among other arguments against this method, their most important finding is that the dye does not permeate in the high-density fibre of rhino horn. The dye remains in the drilling hole so that the horns can easily be cleaned and traded.

Dehorning rhinos for their own safety relies on the assumption that hornless rhinos are of no interest to poachers and will be left alone. Rhinos are tranquilized and the horn is removed with a saw. Despite some successes, there are several cases that suggest dehorning does not actually protects rhinos [99]. Firstly, poachers kill a dehorned rhino for the remaining uncut, or regrown, part of the horn or to avoid tracking the animal again. Secondly, poachers working in the dark may not realize that the rhino has no horn and kill it regardlessly. Finally, in South Africa, it has been proposed to breed rhinos and harvest their horns for a profit [99]. Advocates of this idea believe that flooding the market with rhino horn will decrease the value and discourage poaching [100]. Milner-Gulland et al. [100] devised a mathematical model to investigate the effectiveness of this approach. They estimated that sustainable dehorning will produce near-optimal profits but not deter poachers. Rhinos use their horn for different evolutionary purposes, for example, to defend their territory by fighting over land and chasing away predators. Therefore, removal of the horn may significantly affect the lives of the rhino and their habitat.

### 4.4. Substitution

Advocates of substitution believe that, when rhino horn can be substituted by other substances that can serve the same goal, this could potentially lead to a decrease in rhino poaching. Opponents argue against substitution [101] because advertising palliative substitutes could potentially maintain myths of rhino horn’s medicinal potency, and make the product more socially desirable and actually enlarge the market for rhino horn products.

Pembient [102] runs synthetic keratin through chemical reactions that turn it into a specific type of keratin protein, which is identical to the one that makes up natural rhino horn. Pembient then adds rhino DNA and 3D-prints the rhino horn. This horn is indistinguishable from natural horn by look and feel and lab tests like spectroscopy and DNA analysis will not reveal its artificiality. The idea is to sell the product to middlemen that turn it into end products, such as rhino-beers, ointments, etc. The company claims that they “can meet the demand for horns at one-eighth the black-market price” [102] and hope that the lower price will put an end to poaching of wild rhinoceros.

### 4.5. Demand Reduction

Worldwide, lots of money has been spent annually on studying and protecting rhinos in the wild, while very little has been spent on addressing the underlying demand for the product that drives poaching [6]. WildAid is an organization that focuses primarily on demand reduction of wildlife goods in Asian countries [6]. They create high quality media that is advertised on many different types of media channels such as TV, social media, billboards, and LCD screens in public places. Through mass media campaigning, they aim to reduce demand for Wildlife products with their slogan “when the buying stops, the killing can too”. WildAid works with ambassadors that consist of over 100 celebrated names from all over the world including oscar winning actors, olympic athletes, sporting icons, famous business leaders, musicians and other prominent figures. In 2013, WildAid launched a three-year campaign to reduce rhino horn demand in China by raising awareness in Vietnam and China of the rhino poaching crisis. WildAid also supports Vietnamese lawmakers in banning rhino horn sales and increasing enforcement efforts there and in China.

## 5. Conclusions

In this paper, we conducted a survey on various anti-poaching efforts that aim to protect wild animals from being poached. We investigated efforts that aim to prevent poaching through diplomatic action, law enforcement, demand reduction, negative reinforcement and even substitution. Poaching is not going to be eradicated soon. In order to stop immediate poaching of animals, we studied the current state of the art in poaching detection technologies. These technologies can aid in the immediate protection of endangered wildlife species. We described requirements for poacher detection technologies and identified research challenges through the surveyed works. Effectively taking up these challenges helps to reduce the severity of animal poaching. None of the surveyed works are sufficient in the protection of endangered species. Recently, the concept of utilizing animal sentinels for detection of poaching has been proposed, and, while we believe that this approach has large potential, many challenges remain open for future research.

Securing large forested areas in rugged terrains against intruders proves to be a daunting challenge. There is a need for new approaches that take up the research challenges and provide better protection against poachers. We have argued that the development of cognitive sensor systems, through the combination of multiple sensor technologies and ML techniques, will be able to tackle the challenges found in effective poaching detection. It is our belief that the ultimate long-term solution for the poaching crisis is to remove the drivers of demand. People in the demanding countries should be educated and made aware of the poaching crisis. Until these kinds of actions take effect, there will be a continuous, urgent need for effective anti-poaching solutions that assure the survival of the rhinoceros and elephant species.

## Figures and Tables

**Table 1:** Summary of requirements.

Techniques	Details
Energy Efficiency	Energy saving capability of an anti-poaching technology is still an open issue
Deployment issues	Running power consumption, stealthiness to the environment, maintainability and its easy of deployment are categorized into deployment issues
Robustness	An Anti-Poaching System (APS) should be robust to at least common technical faults
Scalability	The capability of an APS to seamless integrate additional number of devices with the system
Coverage	The ability of an APS to provide full surveillance coverage of a certain protective region
Ethical and legal	The ability of an APS to deal with moral principles and to be abide by the regulations and laws, especially wildlife conservation laws

**Table 2:** Comparison between sensor technologies.

Sensor Technology	Advantages	Disadvantages	Ref.
Radar	Longer range; does not require direct line of sight; estimate velocity of target; tracking of target	No stealth due to active nature; sensitive to interference, such as precipitation and foliage; intruders can reduce radar signature; expensive	[16,17,18,19,20,21,22,23,24,25]
Magnetic	Detection of metal objects such as cars or weapons	Very short range	[21,26,27,28]
Acoustic	Long range; economical	Different acoustic characteristics found in different environments; large vocal repertoire	[26,29,30,31]
Ultrasonic	Long range; economical	Ultrasonic sound is easily absorbed by clothing and foliage	[32]
Optical	Long range; identification of targets	Require line of sight; expensive	[26,33,34,35,36,37,38,39]
Infrared and Thermal	Possibility to detect target at night	Difficult to detect target in hot environments	[36,37,40,41]
Radio Frequency	Does not require line of sight; high level of stealth	Require (buried) cables along perimeter; limited volumetric range	[20,42,43,44,45,46]
Motion	Possibility to classify intrusion type on fences or structures; economical	Range limited to physical structure that sensors are attached to	[41,47,48,49,50,51,52,53]
Seismic	High level of stealth	Range and quality of seismic measurements is different for each environment (soil type)	[25,26,39]
Chemical	Can be used to mark targets for identification; tracking of poached items	Does not prevent animals from being killed; can be obtrusive to animals	[7,32,54,55,56,57]
Animal Sentinels	In theory very large volumetric range; high sensitivity	Many sensors needed; deployment difficulties such as power usage and collaring	[32,58,59,60]

**Table 3:** Overview of poaching detection technologies.

Technique	Advantages	Disadvantages	Ref.
**Perimeter Based Technologies**	Fences are often already in place (sometimes electrified) and can be fortified with the surveyed approaches; some of the surveyed approaches are commercially available	Detect intrusion only along the perimeter of an area, not inside the area itself (linear detection zone). Poachers can enter through the main gate, e.g., disguised as tourist operators.	
Lasers combined with movement detection PIR sensors	Lasers can cover larger distances	No classification; triggered by plants and animals; large False Alarm Rate (FAR)	[41]
Sensor nodes with accelerometers attached to a fence	Classification of intrusion event, thus lower FAR	Many sensors needed; low stealth	[47,48]
Microphonic cables attached to fence	Classification and localization of intrusion	200 m segments; a lot of infrastructure needed; low stealth	[49,50,51,52,53]
Optical fiber attached to fence	Classification and localization of intrusion; segments up to 1000 m; no power needed along segments; insensitive to electromagnetic inference; very sensitive; reliable	Expensive; low stealth.	[50]
Buried optical fiber to detect footsteps	High stealth; harder to destroy; segment ranges up to 10 km	Difficult to bury cables in wildlife areas; soil types vary; expensive	[62,63]
Networked sensors of various types (infrared, magnetic, camera) on and around a fence	Higher resilience; some works include distributed algorithms that aim to decrease FAR	Many sensors needed; large overhead	[26,27,28,33,39,40,64,65]
**Ground Based Technologies**	Can detect intruders on larger area; not limited to linear zone.	Any infrastructure placed inside a wildlife area is prone to be damaged by wildlife.	
Buried coaxial cable	High stealth. Field is wider than optical fiber approach. Commercially available.	Difficult to bury cables in wildlife areas; soil types vary; expensive; volumetric range is not very high.	[20,42,43,44,45]
Fixed sensor node placement with various sensors (RADAR, microphone, light intensity, magnetometers)	Improved animal tracking	Many sensors needed; deployment difficulties such as power usage and destruction of nodes	[21,23,35,66]
Recording animal sounds	Some animals can be heard 4 km away; thus larger range	More challenging approach because: necessary to understand animal sounds; different acoustic characteristics are found in different environments; difference in the vocal repertoire between different species	[30]
Gunshot detection	Gunshots can be heard from far away; thus larger range	High chance of animal being killed before poacher detection	[29]
Ultra Wide Band (UWB) WSN	Classification of intrusion event; thus lower FAR; higher stealth. Improved detection in forested areas.	Limited range; many sensors needed; deployment difficulties such as power usage and destruction of nodes	[67,68]
**Aerial Based Technologies**	Very agile and can cover large areas	Aerial based techniques are obtrusive to habitants and tourists; crashing drones can be a hazard to people and wildlife; can be vulnerable to shooting	
Drones with heat sensing and camera equipment	Works up to 180 m height, thus large range	Unable to detect people under foliage; high running costs	[36,37,69]
Using predictive analytics for automated air surveillance	Improved surveillance accuracy; less sensors needed	Unable to detect people under foliage; high running costs	[70,71]
**Animal Tagging Technologies**	Can potentially cover very large areas with high sensitivity	Many sensors needed; deployment difficulties such as power usage and collaring	
Attach various sensors (cameras, motion, GPS) to animals and classify anomalous behavior	Timely notification of anomalies	Difficult to classify anomalies	[58,59,60]
Monitor physiological status of rhino and implement camera + GPS in rhino horn	Timely notification of animal distress or death; possibility to identify poachers through photos taken from the horn	Still high chance of animal being killed; location data of rhinos is very valuable and can motivate corruption	[34,72]
Detect horn separation from body through RFID	Helps to notify rangers as soon as animal is poached and increases possibility of the poacher’s capture	Rhino will be killed; the RFID chips will grow out of the horn	[73]

PIR, passive infrared; FAR, false alarm rate; RADAR, radio detection and ranging; UWB, ultra wide band; GPS, global positioning system; RFID, radio-frequency identification.
